# Mental Health Status of Female Sex Workers Exposed to Violence in Yangon, Myanmar

**DOI:** 10.1177/10105395221083821

**Published:** 2022-03-10

**Authors:** Yuki Kanayama, Hiroyuki Yamada, Kanako Yoshikawa, Kyaw Wai Aung

**Affiliations:** 1 Department of Economics, Stockholm School of Economics, Stockholm, Sweden; 2 Faculty of Economics, Keio University, Tokyo, Japan; 3 Osaka School of International Public Policy, Osaka University, Toyonaka, Japan; 4 STI Myanmar University, Yangon, Myanmar

**Keywords:** mental health, Myanmar, sex workers, violence

## Abstract

Female sex workers (FSWs) are at considerable risk of developing mental disorders due to the potential for violence associated with their work. However, few studies have comprehensively investigated the types of violence and their impact on the mental health of FSWs. Using data collected from 403 FSWs in Yangon, Myanmar, we investigated how various types of violence perpetrated by clients, employers, and partners affect the severity of mental disorders (anxiety and depression) among FSWs. Our results indicate that economic violence perpetrated by clients and threats of violence from partners induce severe symptoms of anxiety and depression. Furthermore, sexual and economic violence perpetrated by employers results in severe symptoms of anxiety.

## What We Already Know

- Female sex workers (FSWs) are particularly susceptible to mental disorders.
- One of the reasons for this susceptibility is the potential for violence against FSWs.

## What This Article Adds

- This article examines who commits what types of violence and how each type affects various aspects of the mental health of FSWs.
- FSWs who have experienced economic violence from clients experience more severe anxiety and depression compared with those who have not, and sexual violence from employers exacerbates anxiety among FSWs.
- Threats of violence from partners worsen the mental health of FSWs, leading to severe symptoms of anxiety and depression.

## Introduction

Female sex workers (FSWs) are extremely vulnerable to mental disorders. Previous studies have shown that 50% to 75% of FSWs have a mental disorder.^1-3^ The most common symptoms reported by FSWs were those associated with depression and anxiety.^1,4^ Consequently, FSWs are more likely to attempt suicide; for instance, approximately 10% of FSWs in China have attempted suicide in the past 6 months.^5^ New entrants to this profession are particularly at risk of suicide.^6^ This underscores the importance of tackling mental health issues among FSWs and the need to improve social conditions so that women do not feel compelled to enter this profession.

Against this backdrop, the causes of mental disorders in FSWs have been attracting increasing attention among researchers and psychiatrists. Numerous studies have indicated that the potential for violence is one determinant of the mental health of FSWs.^4,7-11^ First, violence perpetrated by FSWs’ partners is strongly associated with mental disorders in FSWs; those who experience violence at the hand of an intimate partner are more likely to experience depression than those who do not.^12^ Second, work-related violence poses a potential threat to the mental health of FSWs.^13^ However, the work environment for FSWs is often unsafe, and their employers frequently act violently toward them, negatively impacting their mental health.^14,15^ Finally, violence perpetrated by clients is exceedingly common; half of FSWs who work on the streets and a quarter of those who work indoors (e.g., at brothels) report that they have experienced violence from clients.^16^ In Gambia, 29% of FSWs have been forced to have sex by their clients.^17^ Unquestionably, violence against FSWs is widely perpetrated by clients, employers, and partners, and this violence places considerable strain on their mental health.

Today, commercial sex remains a vital source of income for many women in lower-income countries. The potential for violence associated with commercial sex constitutes an immediate threat. However, despite the relevance of violence in the mental health of FSWs, little is known about how various types of violence affect the mental health of FSWs. For instance, economic violence (i.e., when clients or employers do not pay the agreed amount of money for the FSW’s services) and sexual violence (when people force FSWs to have sex) affect the mental health of FSWs differently. Moreover, although many studies have focused on depression, this alone is not sufficient to fully understand how extensively violence affects mental health. Certain types of violence may affect different aspects of mental health without inducing depression, and this has not been addressed by existing studies. Therefore, to comprehensively understand the nature of potential violence against FSWs and how to effectively prevent it, the types of violence and their associations with the mental health of FSWs should be examined in more detail.

Our study complements the growing literature on the relationship between mental disorders and potential violence among FSWs. However, it remains unclear who commits what types of violence and how each type of violence affects various aspects of the mental health of FSWs. Our study contributes to the literature by describing in detail how violence affects the mental health of FSWs, thereby opening the door to potential solutions for preventing mental health issues in FSWs.

Yangon, the largest city in Myanmar, has a population of 5 million. Although commercial sex has been prohibited since 1949, it remains a source of income for many poor women. A survey by the Ministry of Health and Sports estimated that there were 7160 FSWs in Yangon as of 2015.^18^ Another study estimated the number of FSWs in Yangon to be approximately 5000 in 2015.^19^ We investigated the nature of violence experienced by FSWs in Yangon and the mental disorders that developed as a result. The results show the extent to which different types of violence perpetrated by clients, employers, and partners affect the severity of mental health issues faced by FSWs.

## Methods

### Data Collection

In July 2019, we conducted a detailed survey of FSWs in Yangon, Myanmar (The description of data collection in this subsection is based on Aung,^20^ Yamada et al,^21^ and Yamada et al.^22^). Due to the nature of the profession, a snowball (nonrandom) sampling method was used.^23,24^ To collect detailed information about FSWs, we recruited five FSW enumerators to conduct interviews with FSWs at six different locations: FSWs’ network offices, drop-in centers, workers’ homes, interviewers’ homes, karaoke clubs, and massage parlors. The five enumerators, who are also representatives of the study group of interest, were intentionally selected as the initial contacts for snowball sampling. At the time of the survey, four of the enumerators were working as FSWs, and one had retired. All of the enumerators belonged to the Sex Workers in Myanmar (SWIM) network, have detailed knowledge about sex work, and can easily contact their peers in Yangon. Because using representative enumerators is considered effective for recruiting members of hidden populations,^25^ we asked the enumerators to contact acquaintances within their network to explain this study, check the availability of these acquaintances, and if possible, arrange face-to-face interviews. To protect the privacy and safety of FSWs and enumerators, we ensured that nobody would enter the room where the interview was being conducted. The survey team reached out to 403 FSWs. All of them agreed to take part in this research project; hence, the response rate was 100%.

We conducted a presurvey to finalize the questionnaire. The enumerators attended a training session before the presurvey as well as a follow-up training session before the actual survey was conducted in order to learn how to collect unbiased information from the respondents. We supervised the enumerators throughout the data collection phase.

The enumerators asked each FSW a set of questions. We were particularly interested in gathering self-reported experiences of anxiety and depression in the past 2 weeks. For anxiety, FSWs were asked how often they had felt nervous, anxious, or on edge in the past 2 weeks. For depression, they were asked how often they had felt depressed or hopeless over the last 2 weeks. For both of these questions, FSWs were asked to choose from the following four options: (1) Not at all, (2) Several days, (3) More than half of the days, or (4) Nearly every day.

We subsequently collected detailed information about the violence experienced by the FSWs, which is the main topic of interest for our research. Our data capture the experiences of FSWs in detail in two respects: who perpetrated the violence against them (clients, employers, or partners) and what types of violent acts the perpetrators committed (physical, sexual, or economic violence or threats of such violence). For instance, we inquired whether the employer committed physical violence against the respondent. In total, we had 12 binary variables indicating whether the respondent experienced each type of violence from each type of person around her, which took a value of 1 for a response of “yes” and 0 otherwise. This detailed classification of violence is a strong advantage of our data set.

We also collected data on their age, education levels, and earnings from commercial sex in the past 30 days. The educational attainment of FSWs was categorized into seven levels: No Official Education, Primary Dropout, Primary Graduate, Secondary Dropout, Secondary Graduate, Higher Education, and University Graduate. Finally, after each interview, the enumerators were asked to subjectively evaluate the appearance of each respondent on a 5-point scale—Not Beautiful at All, Not Beautiful, Standard, Attractive, and Very Attractive—with 1 being the worst and 5 being the best. The descriptive statistics are presented in Table 1.

**Table 1.** Descriptive Statistics.

Variable	Observations	Mean	SD	Minimum	Maximum
Dependent variables					
Anxiety					
1. Not at all (Anxiety)	403	0.720		0	1
2. Several days (Anxiety)	403	0.084		0	1
3. More than half the days (Anxiety)	403	0.127		0	1
4. Nearly every day (Anxiety)	403	0.069		0	1
Depression					
1. Not at all (Depression)	403	0.727		0	1
2. Several days (Depression)	403	0.109		0	1
3. More than half the days (Depression)	403	0.112		0	1
4. Nearly every day (Depression)	403	0.052		0	1
Variables regarding experience of violence					
Physical violence (Clients): 1: Yes, 0: No	403	0.089	Binary	0	1
Sexual violence (Clients): 1: Yes, 0: No	403	0.136	Binary	0	1
Threats of violence (Clients): 1: Yes, 0: No	403	0.094	Binary	0	1
Economic violence (Clients): 1: Yes, 0: No	403	0.191	Binary	0	1
Physical violence (Employers): 1: Yes, 0: No	225	0.022	Binary	0	1
Sexual violence (Employers): 1: Yes, 0: No	225	0.036	Binary	0	1
Threats of violence (Employers): 1: Yes, 0: No	225	0.098	Binary	0	1
Economic violence (Employers): 1: Yes, 0: No	225	0.142	Binary	0	1
Physical violence (Partners): 1: Yes, 0: No	282	0.135	Binary	0	1
Sexual violence (Partners): 1: Yes, 0: No	282	0.092	Binary	0	1
Threats of violence (Partners): 1: Yes, 0: No	282	0.103	Binary	0	1
Economic violence (Partners): 1: Yes, 0: No	282	0.106	Binary	0	1
Control variables					
Age (years old)	403	28.199	7.357	16	48
Education					
1. No official education	403	0.060		0	1
2. Primary dropout	403	0.213		0	1
3. Primary graduate	403	0.144		0	1
4. Secondary dropout	403	0.266		0	1
5. Secondary graduate	403	0.228		0	1
6. Higher education	403	0.074		0	1
7. University graduate	403	0.015		0	1
Beauty					
1. Not beautiful at all	403	0.002		0	1
2. Not beautiful	403	0.057		0	1
3. Standard	403	0.380		0	1
4. Attractive	403	0.511		0	1
5. Very attractive	403	0.050		0	1
log (salary): Earnings from the FSW job in the past 30 days (log-transformed)	403	12.777	0.523	11.0021	13.99783

Abbreviation: FSW, Female sex worker.

### Empirical Strategy

We performed quantitative data analysis to demonstrate how violence affects the mental health of FSWs. We used ordered logit estimation because the dependent variables, Anxiety and Depression , are ordinal. Specifically, we used the following formula:

$$\begin{array}{l} y_{i}^{*}=\beta_{0}+\overset{4}{\underset{k=1}{\sum }}\beta_{1k}v_{ki}+\beta_{2}age_{i}+\beta_{3}edu_{i}\\ +\beta_{4}beauty_{i}+\beta_{5}log\left(salary_{i}\right)+\varepsilon_{i}, \end{array}$$

where i denotes an individual FSW and $y_{i}^{*}$ is a latent variable. v denotes her experience of violence, and k denotes the type of violence. Although we cannot observe $y_{i}^{*}$ , we can instead observe only the categories of response:

$$m_{i}=\{\begin{array}{c} \begin{array}{c} 1ify_{i}^{*}\leq \mu_{1}\\ 2if\mu_{1}<y_{i}^{*}\leq \mu_{2} \end{array}\\ \begin{array}{c} 3if\mu_{2}<y_{i}^{*}\leq \mu_{3}\\ 4if\mu_{3}<y_{i}^{*} \end{array} \end{array},$$

where m denotes the severity of the mental disorders of i . β s and µs are parameters to be estimated.

The severity of mental disorders m consists of two variables (i.e., Anxiety and Depression ), both of which are based on the answers given by the FSWs. As discussed in the “Data Collection” section, these variables were measured on a 4-point scale, with a higher value indicating more severe symptoms. Furthermore, in the interview, the respondents were asked about their experience of violence v , specifically whether they had experienced each of the four types ( k=1,…,4 ) of violence (i.e., physical, sexual, and economic violence and threats of violence) and who committed the violent act (i.e., clients, employers, and partners). These responses help us understand who perpetrated which type of violence against the FSWs. Depending on the relationship between the perpetrators and the FSWs, we included four binary variables indicating whether each of the four types of violence was committed. As mentioned in “Data Collection” section, the following control variables were also included in the model: age, education level, attractiveness (as assessed by enumerators), and earnings from commercial sex (log-transformed).

In total, there are six models for exploring the relationship between violence and mental health (i.e., two mental health conditions × three sources of violence). For each of the 6 models, we show the results both with and without the control variables.

The sample size used for estimation varies: 403 (full sample) for cases of violence perpetrated by clients, 225 for employers, and 282 for partners. This is because most FSWs have no employers and find clients on the streets, online, and at clubs. Moreover, a considerable number of FSWs reported having no partners.

This study was performed in accordance with the principles of the Declaration of Helsinki and its later amendments. Approval was granted by The Ethics Committee of the School for Healthcare Practice, University of Bedfordshire (No: STI011-6-2019-3-2).

## Results

In this section, we present our main results. We investigate whether FSWs who experience violence suffer from more severe mental disorders (i.e., anxiety and depression) than those who do not. The results in Table 2 (anxiety) and Table 3 (depression) show that the mental health of FSWs deteriorates when they experience violence, which is in line with the results of previous studies. The most important point is that the types of violence committed are essential for understanding the association between violence and the mental health of FSWs. We start with the regressions of Anxiety on the variables mentioned above. Table 2 presents the results.

**Table 2.** The Severity of Anxiety and Violence (Ordered Logit Estimation).

Independent variables	Dependent variable: Anxiety					
	(1)	(2)	(3)	(4)	(5)	(6)
Physical violence (Clients)	0.235 (0.490)	0.229 (0.502)				
Sexual violence (Clients)	0.207 (0.449)	0.211 (0.455)				
Threats of violence (Clients)	0.504 (0.458)	0.529 (0.463)				
Economic violence (Clients)	0.995*** (0.302)	0.928*** (0.307)				
Physical violence (Employers)			1.042 (0.644)	0.874 (0.682)		
Sexual violence (Employers)			3.130** (1.319)	3.243** (1.279)		
Threats of violence (Employers)			–0.575 (0.851)	–0.711 (0.840)		
Economic violence (Employers)			1.023* (0.600)	1.215** (0.595)		
Physical violence (Partners)					–0.435 (0.550)	–0.549 (0.621)
Sexual violence (Partners)					1.059* (0.585)	1.137* (0.674)
Threats of violence (Partners)					1.443*** (0.522)	1.518*** (0.557)
Economic violence (Partners)					0.576 (0.497)	0.605 (0.518)
Control variables	No	Yes	No	Yes	No	Yes
Observations	403	403	225	225	282	282

Robust standard errors are in parentheses.

* *P* < .1. **P < .05. ***P < .01.

**Table 3.** Severity of Depression and Violence (Ordered Logit Estimation).

Independent variables	Dependent variable: Depression					
	(1)	(2)	(3)	(4)	(5)	(6)
Physical violence (Clients)	0.515 (0.490)	0.556 (0.479)				
Sexual violence (Clients)	0.0335 (0.432)	0.0735 (0.406)				
Threats of violence (Clients)	0.702 (0.459)	0.621 (0.437)				
Economic violence (Clients)	0.911*** (0.310)	0.892*** (0.294)				
Physical violence (Employers)			0.694 (1.184)	0.494 (1.244)		
Sexual violence (Employers)			1.790 (1.171)	1.900 (1.189)		
Threats of violence (Employers)			0.116 (0.925)	0.178 (0.890)		
Economic violence (Employers)			0.872 (0.549)	0.942* (0.547)		
Physical violence (Partners)					–0.175 (0.525)	–0.253 (0.591)
Sexual violence (Partners)					0.404 (0.670)	0.269 (0.714)
Threats of violence (Partners)					1.931*** (0.565)	1.916*** (0.573)
Economic violence (Partners)					0.380 (0.535)	0.511 (0.544)
Control variables	No	Yes	No	Yes	No	Yes
Observations	403	403	225	225	282	282

Robust standard errors are in parentheses.

* *P* < .1. **P < .05. ***P < .01.

Table 2 highlights the importance of understanding who commits what type of violence. First, economic violence perpetrated by clients is associated with more severe symptoms of anxiety among FSWs, which is statistically significant at the 1% level. Specifically, when clients do not pay the agreed amount of money for services, it causes FSWs to experience anxiety more frequently. Second, sexual violence from employers is associated with even higher levels of anxiety in FSWs, which is statistically significant at the 5% level. Controlling for other factors, if an FSW is forced to have sexual intercourse with her employer, she experiences more severe anxiety. Furthermore, when controlling for individual characteristics (Column 4), economic violence perpetrated by employers intensifies symptoms of anxiety in FSWs. In other words, when an FSW does not receive the agreed amount of salary from her employer, she experiences more severe anxiety. In fact, in Column 4, the coefficient on *Economic Violence (Employers)* is statistically significant at the 5% level. Finally, anxiety in FSWs is associated with threats of violence from partners, which is statistically significant at the 1% level. This means that when an FSW is threatened or intimidated by her partner, the experience is associated with more severe symptoms of anxiety. In summary, economic violence from clients, sexual and economic violence from employers, and threats of violence from partners are associated with more severe symptoms of anxiety in FSWs.

Table 3 shows the results of the ordered logit estimation, which demonstrate the relationship between violence and the severity of depression. The results in Table 3 differ slightly from those in Table 2.

Like Table 2, Table 3 shows that economic violence from clients and threats of violence from partners are associated with symptoms of depression in FSWs. Their coefficients are positive and statistically significant at the 1% level. However, unlike Table 2, Table 3 suggests that violence from employers is not associated with the severity of depression in FSWs (Columns 3 and 4). Thus, we can conclude that, although economic violence from clients and threats of violence from partners are associated with symptoms of depression in FSWs, violence from employers may have little association with their symptoms.

## Discussion

Our results suggest that various types of violence perpetrated by different people affect the mental health of FSWs in different ways. First, those who have experienced economic violence from clients experience more severe anxiety and depression compared with those who have not. Economic violence perpetrated by clients might be of importance in understanding the unequal relationship between FSWs and their clients because of the association with the price negotiation between them. Economic violence perpetrated by clients might occur when price negotiations, which usually take place informally, do not go well. When FSWs feel that they have not received adequate compensation for their services, they may subsequently experience anxiety and/or depression. Second, sexual violence perpetrated by employers exacerbates anxiety in FSWs. FSWs are in economically vulnerable positions relative to their employers, which often leads to abuse by their employers, including sexual abuse (In response to the question “What is your main reason for continuing to work as an FSW?,” about 97% of the participants cited economic reasons [help their family financially, 89%; no other job opportunities, 8%]). FSWs who work in this type of environment may feel mentally trapped and experience severe anxiety. Finally, threats of violence from partners can worsen the mental health of FSWs, leading to more severe symptoms of anxiety and depression. Being an FSW may negatively impact the relationship with their partner because this profession may be neither preferable nor comfortable for the partners. Therefore, the association between violence and the mental health of FSWs varies depending on the type of violence and the aspect of their mental health.

A survey of the literature^4^ identified 17 studies that found associations between experiencing violence and poor mental health in FSWs in low- and middle-income countries, which is broadly consistent with our findings. However, no studies to date have investigated the relationship between potential violence and the mental health of FSWs as comprehensively as the present study, which considered three sources of violence and four types of violence and their influence on two mental health conditions. Consequently, a strict comparison of our findings with those from other studies would be difficult because, to our knowledge, there has been no other comprehensive survey-based study on the link between potential violence and the mental health of FSWs.

More studies regarding the relationship between potential violence and the mental health of FSWs in the contexts of various countries are needed. However, our findings can be interpreted intuitively, so they might apply to some extent to the contexts of neighboring countries.

This study has some limitations. First, due to the nature of the profession, the employed sampling method (snowball sampling) is far from perfect. So, there is a concern regarding internal validity. Second, although our findings are intuitively interpretable, external validity is also an issue because the survey was conducted only in Yangon.

## Conclusion

This study contributes to the literature primarily by demonstrating the extensive associations between violence and the mental health of FSWs. In particular, it reveals who commits what types of violence against FSWs and how it affects their mental health. An improved understanding of this aspect is essential for effectively tackling violence against FSWs and mitigating mental disorders in this population.

Because our study shows the strong associations between violence and mental health in FSWs in more detail compared with other studies, it provides profound insights into the challenges faced by FSWs. The findings of this study should help psychiatrists and other specialists offer targeted assistance and mitigate the impact of potential violence against FSWs.

## References

[1] Puri N Shannon K Nguyen P Goldenberg SM . Burden and correlates of mental health diagnoses among sex workers in an urban setting. BMC Womens Health. 2017;17(1):133. doi:10.1186/s12905-017-0491-y. 29258607 PMC5735638

[2] Zehnder M Mutschler J Rössler W Rufer M Rüsch N . Stigma as a barrier to mental health service use among female sex workers in Switzerland. Front Psychiatry. 2019;10:32. doi:10.3389/fpsyt.2019.00032. PMC6370716 30804819

[3] Ranjbar F Sadeghi-Bazargani H Pishgahi A , et al. Mental health status among female sex workers in Tabriz, Iran. Arch Women Ment Health. 2018;22(3):391-397. doi:10.1007/s00737-018-0907-1. 30128846

[4] Beattie TS Smilenova B Krishnaratne S Mazzuca A . Mental health problems among female sex workers in low- and middle-income countries: a systematic review and meta-analysis. PLoS Med. 2020;17(9):e1003297. doi:10.1371/journal.pmed.1003297. PMC7491736 32931504

[5] Hong Y Fang X Li X Liu Y Li M Tai-Seale T . Self-perceived stigma, depressive symptoms, and suicidal behaviors among female sex workers in China. J Transcult Nurs. 2009;21(1):29-34. doi:10.1177/1043659609349063. 19820172 PMC8185878

[6] Vaniprabha GV Madhusudhan S . Suicide attempts and pattern among the beginners and established female commercial sex workers. J Psychosexual Health. 2019;1(2):140-142. doi:10.1177/2631831819849726.

[7] Abelson A Lyons C Decker M , et al. Lifetime experiences of gender-based violence, depression and condom use among female sex workers in Cameroon. Int J Soc Psychiatry. 2019;65(6):445-457. doi:10.1177/0020764019858646. 31234685

[8] Rössler W Koch U Lauber C , et al. The mental health of female sex workers. Acta Psychiatr Scand. 2010;122(2):143-152. doi:10.1111/j.1600-0447.2009.01533.x. 20105147

[9] Krumrei-Mancuso EJ . Sex work and mental health: a study of women in the Netherlands. Arch Sex Behav. 2016;46(6):1843-1856. doi:10.1007/s10508-016-0785-4. 27439600

[10] Sagtani RA Bhattarai S Adhikari BR Baral D Yadav DK Pokharel PK . Violence, HIV risk behaviour and depression among female sex workers of eastern Nepal. BMJ Open. 2013;3(6):e002763. doi:10.1136/bmjopen-2013-002763. PMC3686218 23794589

[11] Patel SK Saggurti N Pachauri S Prabhakar P . Correlates of mental depression among female sex workers in Southern India. Asia Pac J Public Health. 2015;27(8):809-819. doi:10.1177/1010539515601480. 26307144

[12] Hong Y Zhang C Li X Liu W Zhou Y . Partner violence and psychosocial distress among female sex workers in China. PLoS ONE. 2013;8(4):e62290. doi:10.1371/journal.pone.0062290. PMC3633849 23626798

[13] Ling DC Wong WCW Holroyd EA Gray SA . Silent killers of the night: an exploration of psychological health and suicidality among female street sex workers. J Sex Marital Ther. 2007;33(4):281-299. doi:10.1080/00926230701385498. 17541848

[14] Suresh G Furr LA Srikrishnan AK . An assessment of the mental health of street-based sex workers in Chennai, India. J Contemp Crim Justice. 2009;25(2):186-201. doi:10.1177/1043986209333590.

[15] Duff P Sou J Chapman J , et al. Poor working conditions and work stress among Canadian sex workers. Occup Med (Oxf). 2017;67(7):515-521. doi:10.1093/occmed/kqx092. 29016896 PMC6317264

[16] Church S . Violence by clients towards female prostitutes in different work settings: questionnaire survey. BMJ. 2001;322(7285):524-525. doi:10.1136/bmj.322.7285.524. 11230067 PMC26557

[17] Sherwood JA Grosso A Decker MR , et al. Sexual violence against female sex workers in the Gambia: a cross-sectional examination of the associations between victimization and reproductive, sexual and mental health. BMC Public Health. 2015;15(1):270. doi:10.1186/s12889-015-1583-y. PMC4375842 25886187

[18] National AIDS Programme. Myanmar integrated biological and behavioural surveillance survey and population size estimates among female sex workers (FSW). Ministry of Health and Sports. Published 2019. Accessed February 18, 2022. https://www.aidsdatahub.org/sites/default/files/resource/myanmar-ibbs-and-population-size-estimates-among-pwid-2017-2018.pdf.

[19] Thein ST Aung T McFarland W . Estimation of the number of female sex workers in Yangon and Mandalay, Myanmar. AIDS Behav. 2015;19(10):1941-1947. doi:10.1007/s10461-015-1169-9. 26267254

[20] Aung KW . Violence Among Female Sex Workers and Challenges in Access to Health Care in Yangon Region, Myanmar. Luton, England: Faculty of Health & Social Science, University of Bedfordshire; 2019.

[21] Yamada H Kanayama Y Yoshikawa K Aung KW . Risk attitude, risky behavior, and price determination in the sex market: a case study of Yangon, Myanmar. Keio-IES Discussion Paper Series. Published 2020. Accessed February 18, 2022. https://ies.keio.ac.jp/upload/pdf/en/DP2020-013.pdf.

[22] Yamada H Kanayama Y Yoshikawa K Aung KW . Place premiums: analysis of place-based characteristics of prostitutes in Yangon, Myanmar. Published 2020. doi:10.2139/ssrn.3753889.

[23] Atkinson R Flint J . Accessing hidden and hard-to-reach populations: snowball research strategies. Soc Res Update. 2001;33(1):1-4.

[24] Shaghaghi A Bhopal RS Sheikh A . Approaches to recruiting “hard-to-reach” populations into research: a review of the literature. Health Promot Perspect. 2011;1(2):86-94. doi:10.5681/hpp.2011.009. 24688904 PMC3963617

[25] Ellard-Gray A Jeffrey NK Choubak M Crann SE . Finding the hidden participant: solutions for recruiting hidden, hard-to-reach, and vulnerable populations. Int J Qual Methods. 2015;14(5). doi:10.1177/1609406915621420.

